# Research progress and perspectives of non-coding RNAs in primary biliary cholangitis: from mechanisms to therapeutics

**DOI:** 10.3389/fmed.2025.1611640

**Published:** 2025-08-04

**Authors:** Wangqi Chen, Qinghua Li, Yuxia Xie, Hong Zhu

**Affiliations:** ^1^Department of Gastroenterology, The First Affiliated Hospital of Nanjing Medical University, Nanjing, Jiangsu, China; ^2^Department of Gastroenterology, The First School of Clinical Medicine of Nanjing Medical University, Nanjing, Jiangsu, China

**Keywords:** primary biliary cholangitis, non-coding RNAs, immune dysregulation, biomarker, therapeutic target

## Abstract

Primary biliary cholangitis (PBC), an autoimmune-mediated cholestatic liver disease with a female predominance, remains enigmatic in its pathogenesis despite advances in understanding immune dysregulation, bile acid dyshomeostasis, inflammatory cascades, gut-liver axis crosstalk, and sex-biased mechanisms. Although ursodeoxycholic acid is widely recognized as the first-line therapy, its variable efficacy underscores the need for novel biomarkers and targeted therapies. Non-coding RNAs (ncRNAs), though not encoding proteins, have emerged as promising candidates due to their pivotal regulatory roles in autoimmune processes. This review systematically delineates the interplay between ncRNAs (miRNAs, lncRNAs, circRNAs) and key PBC mechanisms, evaluates their diagnostic and therapeutic potential, and proposes future research frameworks to bridge molecular insights with clinical translation.

## 1 Introduction

Primary biliary cholangitis (PBC), formerly termed “primary biliary cirrhosis”, is a progressive autoimmune liver disease characterized by destructive lymphocytic cholangitis and elevated levels of antimitochondrial antibodies (AMAs), leading to cholestasis, fibrosis, and eventual liver failure (1, 2). Due to the insidious onset of PBC, approximately half of the patients remain asymptomatic during the initial stages or present solely with non-specific symptoms such as fatigue and pruritus. Left untreated, the disease may progress relentlessly to cirrhosis and hepatocellular carcinoma in advanced cases (3). Although traditionally considered a rare disorder, accumulating epidemiological evidence reveals an increasing global prevalence of PBC across diverse ethnic populations, imposing growing clinical burdens, particularly among middle-aged demographics (4, 5). Notably, while female predominance persists (current female-to-male ratio < 5:1), recent surveillance data indicate a progressive rise in male incidence rates (6). Ursodeoxycholic acid (UDCA), a well-studied bile acid (BA) regulator, is the only first-line medication that has achieved FDA approval and clinical utilities, while a proportion of patients exhibit suboptimal responses (7). Although second-line agents, including obeticholic acid (OCA) and bezafibrate, have shown complementary effects when combined with UDCA, their capacity to fundamentally alter disease trajectory remains limited, as evidenced by marginal improvements in long-term prognostic outcomes (8, 9). This therapeutic impasse stresses the imperative for developing precision diagnostic tools and mechanism-driven therapies, which hold promise for achieving clinically meaningful endpoints and substantially improving the quality of life in PBC patients.

The pathogenesis of this disorder involves a complex interplay of genetic predisposition, environmental triggers, and other contributing factors (1). Key mechanisms involved in the progression of PBC include immune dysregulation, abnormal BA metabolism, and inflammatory response (10, 11). Recent advances further implicate gut microbiota-derived metabolites and exosomes in modulating metabolism and promoting hepatocellular carcinoma (12). However, a critical knowledge gap remains: how these disparate pathways are coordinated at the molecular level to drive disease progression.

Non-coding RNAs (ncRNAs) comprise a diverse class of RNA molecules that lack protein-coding capacity, yet critically regulate cellular processes through intricate molecular mechanisms (13). This family encompasses three principal subtypes: microRNAs (miRNAs, 19–25 nucleotides), long non-coding RNAs (lncRNAs, > 200 nucleotides), and circular RNAs (circRNAs) characterized by covalently closed structures (14). Emerging evidence reveals that ncRNAs orchestrate gene expression networks by interacting with DNA, RNA, and proteins, thereby governing transcriptional programs, translational efficiency, and post-translational modifications (15). miRNAs can function as post-transcriptional gene regulators by either cleaving specific mRNAs or inhibiting mRNA translation, whereas lncRNAs – typically featuring a 5′-terminal 7-methylguanosine cap and 3′-polyadenylation – exhibit nuclear, cytoplasmic, or organelle-specific localization to modulate chromatin remodeling, RNA splicing, and protein interactions (16, 17). In contrast, circRNAs evade exonuclease degradation through their closed-loop configuration formed through covalent back-splicing, conferring exceptional stability and context-dependent regulatory functions (18, 19). Of particular relevance to autoimmunity, ncRNAs serve as master regulators of immune homeostasis by fine-tuning immune cell differentiation, cytokine production, and tolerance mechanisms (20). Dysregulated ncRNA expression profiles have been mechanistically linked to multiple autoimmune disorders, including rheumatoid arthritis (RA), systemic lupus erythematosus (SLE), and PBC (20). Notably, ncRNAs can be encapsulated and transported within exosomes to generate stable “exosomal ncRNAs” that mediate intercellular communication and pathophysiological signaling across tissues (21, 22).

This review synthesizes cutting-edge evidence on ncRNA dysregulation in PBC to delineate how miRNAs, lncRNAs, and circRNAs orchestrate PBC pathogenesis across immune, metabolic, and microbiotic axes. Based on these advances, we critically evaluate their emerging roles as diagnostic biomarkers and therapeutic targets. By bridging molecular insights with translational applications, this work aims to catalyze ncRNA-based innovations for personalized PBC management.

## 2 Mechanistic nexus: how ncRNAs drive PBC pathogenesis

**FIGURE 1:**
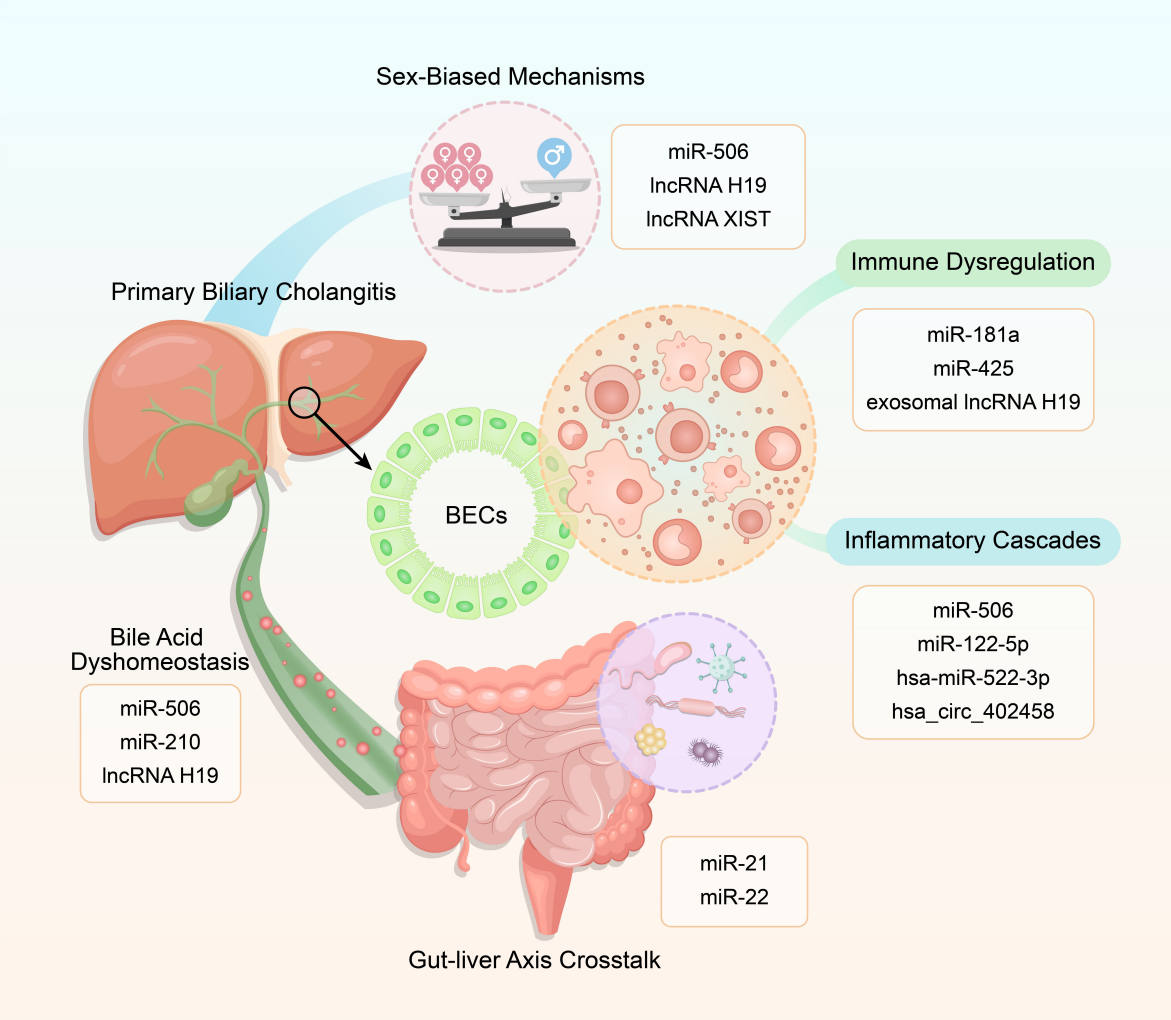
Mechanistic interplay of non-coding RNAs (ncRNAs) in primary biliary cholangitis (PBC) pathogenesis. This schematic illustrates five core pathways – immune dysregulation, bile acid dyshomeostasis, inflammatory cascades, sex-biased mechanisms, and gut-liver axis crosstalk – each modulated by specific ncRNAs (e.g., miRNAs, lncRNAs, circRNAs).

Non-coding RNAs orchestrate PBC pathogenesis through interconnected circuits governing immune tolerance collapse, BA dyshomeostasis, inflammatory cascades, sex-biased epigenetic remodeling, and gut-liver crosstalk, as visualized in Figure 1, forming a self-amplifying network that bridges molecular dysregulation to clinical progression.

### 2.1 Immune dysregulation

The immunopathogenesis of PBC is characterized by aberrant immune cell infiltration within portal tracts and periductal regions, with dysregulated lymphocyte subsets driving bile duct epithelial cell (BEC) destruction (23). T lymphocytes emerge as central orchestrators, where CD8+ cytotoxic T cells specifically target BECs, while CD4+ T cells amplify immunopathology via cytokine hypersecretion (e.g., IL-12, IFN-γ) and B cell activation (24–26). Notably, the destabilization of immune homeostasis is exacerbated by quantitative and functional deficiencies in regulatory T cells (Tregs), coupled with Th17 cell expansion, resulting in a skewed Treg/Th17 ratio that perpetuates autoimmunity (27, 28). B lymphocytes contribute to PBC progression through dual mechanisms: antigen presentation and generation of pathognomonic AMAs (29, 30). Emerging evidence implicates innate immune components, including hyperactivated NK cells with enhanced perforin expression and macrophages exhibiting impaired phagocytosis of apoptotic cholangiocytes, as critical mediators of cholestatic injury (31, 32). Other immune components, including monocytes (33), mast cells (34), and the complement system (35) further compound this immunopathological cascade.

It is worth mentioning that ncRNAs intricately regulate these immune perturbations. miR-181a downregulation in CD4+ T cells promotes PBC progression by suppressing Th17 apoptosis via B-cell lymphoma-2 (BCL-2) upregulation, thereby sustaining T cell hyperactivation (36). Similarly, diminished miR-425 expression in PBC-derived CD4+ T cells correlates with elevated inflammatory cytokine production, potentially mediated through N-Ras hyperactivation in T cell receptor (TCR) signaling pathways (37). These findings posit miRNA restoration therapies as promising strategies to recalibrate T cell dysfunction in PBC (38). lncRNAs exert multimodal immunomodulatory effects, with cholangiocyte-derived exosomal lncRNA H19 exemplifying a novel intercellular communicator that activates macrophages via the chemokine (C-C motif) ligand 2 (CCL-2)/chemokine receptor 2 (CCR-2) signaling, amplifying cholestatic inflammation (39, 40). Beyond canonical ncRNAs, circRNAs introduce an additional regulatory dimension through miRNA sponging and epigenetic modulation, with significant roles in immune cell differentiation and autoimmune activation (41, 42). Despite their theoretical relevance, the miRNA-circRNA interactome in PBC remains underexplored, warranting systematic profiling to delineate its therapeutic potential.

### 2.2 Bile acid dyshomeostasis

Dysregulated BA metabolism represents a pathological hallmark of cholestatic liver diseases, characterized by intrahepatic BA accumulation and elevated systemic BA concentrations. These cytotoxic BA overloads drive cholangiocyte hyperproliferation, premature senescence, and hepatocyte inflammatory cascades, culminating in progressive cholestatic liver injury (43). Anion exchanger 2 (AE2) and the “bicarbonate umbrella” constitute a critical defense mechanism against BA toxicity through bicarbonate secretion maintenance (44). Of particular interest, AE2 dysfunction and bicarbonate barrier impairment develop into consistent features during PBC progression. Current first-line therapy with UDCA exerts therapeutic effects partially through AE2 upregulation and bicarbonate secretion restoration, thereby counteracting BA-induced epithelial damage (45). Beyond direct cytotoxicity, BAs integrate multifaceted signaling networks via the farnesoid X receptor (FXR), modulating metabolic homeostasis, fibrogenesis, and immune regulation. This mechanistic insight has propelled the clinical application of FXR agonists like OCA in PBC management (46).

Emerging evidence implicates ncRNAs as pivotal regulators of BA dyshomeostasis in PBC pathogenesis. Cholangiocyte-specific miR-506 overexpression disrupts biliary bicarbonate secretion by suppressing both Cl-/HCO3- AE2 and inositol 1,4,5-trisphosphate receptor type III (InsP3R3), establishing a direct molecular link between miRNA dysregulation and cholestatic phenotypes (47, 48). The epigenetic dimension of BA regulation is further illustrated by miR-210, which exhibits marked upregulation in cholestatic murine models and PBC patient livers (49). Through targeted inhibition of the histone methyltransferase mixed-lineage leukemia-4 (MLL4), a transcriptional coactivator essential for FXR-mediated BA homeostasis, miR-210 exacerbates BA-induced hepatotoxicity (49).

At the lncRNA level, hepatic H19 overexpression in cholestatic patients correlates with ductular reaction and fibrogenesis, two hallmark pathological processes driven by BA overload. Mechanistic studies reveal that H19 promotes cholestatic fibrosis via dual modulation of epithelial-mesenchymal transition markers: upregulating epithelial cell adhesion molecule (EpCAM) while suppressing zinc finger E-box-binding homeobox 1 (ZEB1) (50). Furthermore, H19-mediated cholestasis involves polypyrimidine tract-binding protein 1 (PTBP1) downregulation and subsequent aberrant let-7 expression, suggesting a multi-layered regulatory network (51). These findings position lncRNA H19 as a central hub integrating epigenetic regulation with BA-induced fibrotic responses.

### 2.3 Inflammatory cascades

Chronic hepatic inflammation constitutes a hallmark of PBC progression, driving fibrotic remodeling through intertwined cell death pathways and cytokine storms (52). The activation of inflammasome-mediated apoptosis in PBC enhances the release of inflammatory factors and promotes the inflammatory response (53). Research has indicated that the initial proinflammatory damage in PBC primarily relies on Galectin-3-mediated NLRP3 inflammasome activation and the subsequent production of proinflammatory cytokines, such as IL-17 and IL-1β (54, 55). Caspase-10 emerges as a critical rheostat, modulating necroptotic and pyroptotic cell death modalities that not only promote hepatobiliary inflammation but also activate hepatic stellate cells (HSCs) to propagate fibrosis (56). Besides, studies have revealed that cell pyroptosis, triggered by caspase-4 and gasdermin D (GSDMD), underlies cholestatic liver failure (57).

Non-coding RNAs intricately regulate these inflammatory circuits through multilayered mechanisms. Erice et al. (58) identified an IL-8/IL-12-driven positive feedback loop wherein proinflammatory cytokines upregulate miR-506 in cholangiocytes via promoter activation. This miR-506 surge impairs mitochondrial energetics through pyruvate dehydrogenase complex E2 dysregulation, creating metabolic-inflammatory crosstalk that perpetuates PBC-like pathology (58). Complementing this finding, Zhang et al. (59) demonstrated that HSC-derived exosomal miR-122-5p suppresses p38 MAPK signaling in intrahepatic biliary epithelial cells (IBECs), effectively dampening inflammatory mediator release. The therapeutic potential of miR-122-5p restoration was highlighted by its capacity to normalize IBEC cytokine profiles *in vitro*. circRNAs further expand this regulatory network, as evidenced by Zheng et al. (60), who identified hsa_circ_402458 as a putative miRNA sponge sequestering hsa-miR-522-3p, a miRNA implicated in chronic inflammation. The differential expression of hsa_circ_402458 in UDCA-treated PBC patients suggests its dual role as both an inflammatory modulator and a potential therapeutic response biomarker (60).

### 2.4 Sex-biased mechanisms

The striking female predominance in PBC presents compelling evidence for sex-specific pathogenic mechanisms. This sexual dimorphism manifests clinically through distinct phenotypic presentations: female patients exhibit a higher prevalence of pruritus and abdominal discomfort compared to males, while male patients demonstrate elevated mortality rates due to delayed diagnosis and challenges in clinical differentiation (61, 62). Accumulating data suggest X chromosome-linked epigenetic regulation and estrogen-responsive ncRNA networks as key contributors to this sex-based disparity (63).

Notably, the miR-506-AE2-sAC axis concept was previously introduced, where X chromosome-derived miR-506 overexpression in PBC cholangiocytes epigenetically suppresses AE2 expression. This suppression triggers soluble adenylate cyclase (sAC) activation, culminating in cholangiocyte apoptosis (64). A hypothesis was subsequently proposed that epigenetic changes linked to the X chromosome during the PBC occurrence result in a female-biased activation of this axis, providing a plausible explanation for the disproportionate female susceptibility to PBC (64). The sexual dimorphism extends to lncRNA networks through similar dual mechanisms (65). Experimental evidence from the multidrug resistance 2 gene knockout [Mdr2(−)] cholestasis model reveals sex-specific lncRNA H19 overexpression in female mice, where estrogen induces its expression through the extracellular signal-regulated kinase 1/2 signaling pathway (66). This estrogen-lncRNA H19 synergy exacerbates hepatobiliary injury in female Mdr2(−) mice (66). X chromosome inactivation (XCI) plays an important role in the distinction of disease susceptibility between males and females. The lncRNA X inactive specific transcript (XIST), involved in regulating XCI, is exclusively expressed by the inactivated X chromosome (67). She et al. (68) reported a correlation between lncRNA XIST and atypical lymphocytes in PBC patients, noting that its overexpression stimulated the proliferation and differentiation of naive CD4+ T cells, potentially driving female-biased autoimmune responses (68). While research in this field is still in its nascent stages, it underscores the potential clinical significance of focusing on gender-specific diagnostic approaches, management strategies, and therapies, in light of the association between lncRNAs and PBC sexual dimorphism.

These findings collectively establish a multilayered regulatory framework where sex chromosome biology intersects with hormone-responsive ncRNA networks to shape PBC pathogenesis. While current research remains exploratory, these mechanistic insights advocate for sex-stratified diagnostic algorithms and therapeutic development.

### 2.5 Gut-liver axis crosstalk

Primary biliary cholangitis pathogenesis is inextricably linked to gut microbial ecosystem collapse, characterized by altered gut microbes, diminished microbial diversity, and impaired intestinal barrier integrity (69). Mendelian randomization analyses by Zhang et al. (70) have confirmed a causal relationship between specific gut microbiota genera (e.g., *Lachnospiraceae_UCG_004*, *Ruminococcaceae*) and PBC susceptibility, highlighting the gut microbiome as a potential disease modifier. Notably, persistent gut microbiota dysbiosis and its metabolites correlate with accelerated fibrosis and suboptimal therapeutic responses in PBC (71). Advanced PBC fibrosis is further associated with elevated fecal short-chain fatty acids (SCFAs) and microbial community shifts, suggesting metabolite-driven crosstalk between the gut and liver (72).

The BA-microbiome axis enhances cholestatic damage through bidirectional interactions: the gut microbiota modulates BA signaling by metabolizing BAs through specific enzymes, while the dynamics of BAs reciprocally shape microbial composition (73). Recent findings highlight ncRNAs as potential mediators of this gut-liver dialog. For instance, miR-21 exacerbates cholestatic liver injury by directly suppressing intestinal *Lactobacillus* populations in bile duct-ligated mice (74). *Lactobacillus* depletion disrupts gut homeostasis, while its supplementation attenuates hepatic fibrosis, mimicking the protective effects observed in miR-21 knockout models (74). This reinforces a mechanistic pathway whereby host-derived miRNAs modulate the gut microbiota to influence liver pathology. Microbial metabolites such as butyrate, an SCFA elevated in advanced PBC fibrosis (72), exert dual roles in liver pathophysiology. Butyrate induces ROS-mediated apoptosis in hepatic cells via the miR-22/SIRT-1 pathway, illustrating how microbiota-derived metabolites can directly regulate ncRNA networks to influence hepatocyte survival (75). Chronic inflammation, a hallmark of PBC, further disrupts gut-liver communication. Multi-omics analyses in IFN-γ-driven murine models reveal sex-biased alterations in gut microbiota and their metabolites, particularly those linked to BA metabolism and nuclear receptor signaling (e.g., FXR) (76). These findings align with clinical observations of dysregulated BA signaling in PBC, suggesting that ncRNAs may interface with inflammatory pathways to perturb microbial-metabolic networks. Unfortunately, the ncRNA-microbiome-BA triad in PBC remains critically underexplored, with a paucity of mechanistic studies delineating their interactions and limited experimental validation of their pathophysiological synergy.

## 3 Diagnostic and therapeutic potential of ncRNAs

Non-coding RNAs are redefining precision medicine in PBC through their dual roles as sensitive disease biomarkers and druggable therapeutic targets, while systems-level network analyses uncover their hierarchical control over immune-metabolic dysregulation, bridging molecular insights to clinical translation.

### 3.1 ncRNAs as biomarkers

The quest for non-invasive diagnostic tools in PBC has been revitalized by ncRNAs, which exhibit remarkable stability in biofluids and disease-specific expression patterns (77). Of particular interest is miR-34a, a p53-regulated transcript implicated in fibrotic pathogenesis across multiple organ systems (78). Pan et al. (79) revealed elevated circulating miR-34a levels in PBC patients, which mechanistically promote hepatic fibrogenesis through transforming growth factor β1 (TGF-β1)/Smad pathway activation and subsequent epithelial-mesenchymal transition (EMT). This molecular cascade positions miR-34a as a novel biomarker for monitoring fibrotic progression in PBC. Comparative analyses reveal distinct miRNA signatures between serological subgroups. AMA-negative PBC patients exhibit significant upregulation of serum miR-21 and miR-150 compared to their AMA-positive counterparts, with concomitant alterations in downstream molecular targets (80). These differential expression patterns suggest potential diagnostic utility for AMA-negative subtype identification and fibrosis staging (80). Furthermore, therapeutic response monitoring studies identified characteristic miRNA profiles in UDCA-resistant patients. Non-responsive individuals display elevated serum levels of miR-122 and miR-378f alongside reduced miR-4311 and miR-4714-3p expression, with these fluctuations demonstrating significant correlations with conventional hepatic function parameters (81). Such miRNA expression patterns may provide clinically relevant biomarkers for assessing therapeutic efficacy in refractory PBC cases.

Beyond miRNAs, lncRNAs and circRNAs have also emerged as potential biomarkers for PBC. Jiang et al. (82) reported that lncRNA H19 is markedly enriched in hepatocyte nuclear factor 4α(HNF4α) (+) periportal hepatocytes within liver samples from female PBC patients, as revealed by a novel technique combining *in situ* hybridization (ISH) with immunofluorescence (IF) co-labeling. lincRNAs, a specific subtype of lncRNAs, have been implicated in various cellular functions and hold promise for elucidating the etiology of PBC (83). Researchers have identified several differentially expressed lncRNAs in the plasma of PBC patients by bioinformatics analyses, including the downregulation of LINC00312 and the upregulation of LINC00472 and LINC01257, suggesting their potential roles in disease progression and utility in diagnosis and staging (84). Additionally, Zheng et al. (60) observed differential expression of hsa_circ_402458 in the plasma of UDCA-treated versus untreated PBC patients, predicting two potential downstream targets (hsa-miR-522-3p and hsa-miR-943). Their findings suggest that hsa_circ_402458 may serve as a promising biomarker for PBC. Collectively, these studies highlight the translational potential of ncRNA biomarkers in revolutionizing PBC management through enhanced diagnostic precision, dynamic disease monitoring, and personalized treatment strategies.

### 3.2 Therapeutic targeting

The current therapeutic paradigm for PBC relies on UDCA as first-line therapy and OCA as second-line intervention. However, persistent challenges remain, including suboptimal biochemical responses in 30%–40% of UDCA-treated patients and dose-limiting pruritus/hepatotoxicity associated with OCA therapy (85, 86). These limitations underscore the urgent need for new therapeutic strategies targeting fundamental pathogenic mechanisms.

Accumulating data position miRNAs as viable therapeutic candidates, primarily due to their critical regulatory roles in anti-cholestatic hepatoprotection and immune modulation. Enoxacin, a small-molecule fluoroquinolone known to enhance miRNA biogenesis, has demonstrated therapeutic potential in murine PBC models by upregulating miRNA expression in CD8+ T cells, thereby attenuating their pathogenicity and preventing autoimmune biliary injury (38). Intriguingly, melatonin exhibits dual regulatory effects on miRNA networks in cholangiocytes, suppressing pro-apoptotic miR-34 while upregulating anti-apoptotic miR-132, suggesting its potential as a cytoprotective agent against PBC-related biliary damage (87). Afonso et al. (88) observed that miR-21 ablation in hepatocytes of a bile duct ligation (BDL) murine model lowered serum liver injury markers and reduced oxidative stress, fibrosis, and hepatocyte degeneration, providing valuable insights into ameliorating cholestasis. Recent advances suggest miR-29a as a potent epigenetic modulator that attenuates hepatic stellate cell activation through dual inhibition of BRD4 and EZH2 signaling pathways, with preclinical studies demonstrating that both miR-29a mimics and BRD4 inhibitor JQ1 effectively suppress fibrogenic gene networks (c-MYC, SNAI1) and cellular proliferation in cholestatic models, positioning miR-29a restoration as a promising therapeutic strategy for hepatic fibrogenesis (89). Clinical observations consistently reveal hepatic peroxisome proliferator-activated receptor α (PPARα) downregulation in PBC patients, a phenomenon mechanistically linked to miR-155 overexpression (90). A key observation is the UDCA-mediated suppression of miR-155, which restores PPARα-dependent homeostasis in inflammation and metabolism, providing compelling evidence for targeting miR-155 in precision medicine (90). Furthermore, therapeutic strategies targeting the BA-Egr-1-Limb expression 1-like protein (LIX1L) axis show translational promise, with both LIX1L inhibition and Adeno-Associated Virus (AAV)-mediated miR-191-3p overexpression exhibiting significant hepatoprotection via suppression of BA synthesis (91).

The therapeutic landscape of lncRNAs and circRNAs is rapidly evolving but remains underexplored in PBC pathogenesis. As previously noted, macrophages play an important part in the etiology of chronic liver disorders. Specifically, selective macrophage clearance has been shown to reduce liver lncRNA H19 expression, inhibiting cholestatic liver injury and fibrosis (92). Exosomal lncRNA H19, derived from cholangiocytes, has been implicated in the aggravation of cholestatic liver fibrosis by enhancing the differentiation and activation of HSCs in cholestatic mice and PBC patients (93). The dual diagnostic and therapeutic potential of lncRNAs in PBC is increasingly recognized, yet translating this promise requires resolving cell-specific mechanistic nuances and optimizing delivery systems. circMTO1 (hsa_circ_0007874) exemplifies the therapeutic duality of circRNAs in hepatic pathologies. In liver fibrosis, it suppresses HSC activation by sponging miR-181b-5p to upregulate PTEN (94), while in hepatocellular carcinoma (HCC), it inhibits tumor progression via miR-9 sequestration and p21 activation, with its downregulation predicting poor patient prognosis (95). While circMTO1 shows promise in liver fibrosis and HCC, its applicability to PBC therapy is currently limited by unresolved mechanistic and translational gaps.

### 3.3 ncRNA-centered regulatory networks

With the advancement of high-throughput technologies, miRNA-centered regulatory networks have emerged as reliable and accurate analytical techniques for elucidating interconnections and regulatory mechanisms in various diseases. Through Gene Ontology (GO) analysis tailored to PBC characteristics, Li et al. (96) identified PFKL as a critical node within the miRNA-target network, demonstrating its regulatory control over glycolytic processes. In cholestatic liver injury research, a comprehensive miRNA-mRNA network was constructed, revealing six pivotal miRNAs (miR-122, miR-30e, let-7c, miR-107, miR-503, and miR-192) and eight core genes (PTPRC, TYROBP, LCP2, RAC2, SYK, TLR2, CD53, and LAPTM5) that predominantly mediate immune-related pathways (97). Notably, SYK was identified as a potential biomarker for predicting UDCA treatment responsiveness in PBC patients, possibly through modulation of complement activation and monocyte dynamics (97). Comparative analysis of the Gene Expression Omnibus (GEO) microarray datasets revealed 34 differentially expressed genes (DEGs) between PBC patients and healthy controls (22 upregulated, 12 downregulated) (98). This genomic landscape enabled the construction of a multi-layered transcription factor-DEG-miRNA regulatory network, with abundant interacting miRNAs involved, such as has-miR-98-5p, has-miR-452-5p, and has-miR-497-5p (98). Within this network, AKR1B10 was nominated as a PBC-critical gene, with clinical evidence demonstrating that its hepatic overexpression correlates with both disease severity and progression to hepatocellular carcinoma (98). These findings collectively underscore the substantial potential of miRNA-based network analysis in PBC research. This systems biology approach not only facilitates the identification of disease-associated genes but also provides novel mechanistic insights into PBC pathogenesis, potentially revealing therapeutic targets for clinical intervention.

Collectively, the emerging roles of ncRNAs in PBC diagnosis, disease stratification, and targeted therapeutic interventions are systematically outlined in Table 1, highlighting their translational potential from mechanistic discoveries to clinical applications.

**TABLE 1:** Comprehensive overview of non-coding RNAs (ncRNAs) with diagnostic and therapeutic potential in primary biliary cholangitis (PBC).

Category	Key molecule(s)	Mechanistic insights	Clinical relevance	References
Biomarkers	miR-34a	Activates TGF-β1/Smad pathway, promoting hepatic fibrogenesis and EMT	Serum biomarker for fibrotic progression monitoring	(79)
	miR-21, miR-150	Upregulated in AMA-negative PBC; modulate downstream targets	Subtype-specific diagnostic markers	(80)
	miR-122, miR-378f, miR-4311, miR-4714-3p	Dynamic association with hepatic function parameters	Predictive biomarkers for therapeutic resistance	(81)
	lncRNA H19	Enriched in HNF4α(+) periportal hepatocytes (female PBC)	Gender-specific diagnostic marker	(82)
	LINC00312, LINC00472, LINC01257	Differentially expressed in plasma	Potential staging/prognostic biomarkers	(84)
hsa_circ_402458	Sponges hsa-miR-522-3p/hsa-miR-943	Distinguishes UDCA-treated vs. untreated patients	(60)
Therapeutic targets	miR-21	Hepatocyte-specific ablation reduces oxidative stress, fibrosis, and degeneration	Ameliorates cholestatic injury in BDL murine models	(88)
	miR-29a	Dual inhibition of BRD4/EZH2 pathways; suppresses fibrogenic networks	Anti-fibrotic therapy (preclinical validation)	(89)
	miR-155	miR-155 suppression by UDCA restores PPARα-mediated homeostasis	Precision therapeutic target (inflammation-metabolism crosstalk)	(90)
	miR-191-3p	Inhibits BA synthesis	AAV-mediated overexpression reduces cholestatic injury	(91)
	lncRNA H19	Reduces cholestatic liver injury and fibrosis	Therapeutic potential via macrophage modulation	(92, 93)
	circMTO1 (hsa_circ_0007874)	Sponges miR-181b-5p in fibrosis; inhibits miR-9 in HCC	Multi-disease therapeutic candidate (limited PBC validation)	(94, 95)
Regulatory Networks	miR-122, miR-30e, let-7c, miR-107, miR-503, miR-192	miRNA-mRNA network; modulates immune pathways	Predictor of UDCA responsiveness (SYK)	(97)
	has-miR-98-5p, has-miR-452-5p, has-miR-497-5p	TF-DEG-miRNA regulatory network	Overexpression correlates with PBC severity and HCC transformation (AKR1B10)	(98)

## 4 Challenges and future directions

Despite growing interest in ncRNAs as pivotal players in PBC, their clinical translation remains hindered by technical ambiguities in mechanistic validation, therapeutic delivery challenges, and untapped diagnostic potential – barriers now being addressed through emerging technologies like single-cell multiomics and CRISPR screening.

### 4.1 Technical and mechanistic limitations

Current studies predominantly focus on miRNA profiling, while the functional characterization of lncRNAs and circRNAs remains rudimentary. Notably, tissue-specificity complicates biomarker discovery: hepatic miR-506 is upregulated in PBC cholangiocytes but undetectable in serum, limiting its clinical utility (58). The field faces additional challenges in circRNA research, where accurate annotation is complicated by overlapping transcript isoforms (e.g., circRNA_0007874 derived from the same host gene as linear transcripts), necessitating advanced methodologies like long-read sequencing or CRISPR/Cas system validation (94). Existing mechanistic investigations are further constrained by reliance on murine models, which fail to fully replicate the complex immune-microenvironment interactions observed in human PBC pathophysiology. Moreover, emerging evidence suggests potential crosstalk between ncRNA networks and gut microbiota dysbiosis, a well-established pathogenic mechanism in PBC (40). However, the regulatory mechanisms through which ncRNAs might modulate gut microbiota-mediated effects on disease progression remain unexplored, highlighting a critical knowledge gap that warrants systematic investigation in future studies.

### 4.2 Translational hurdles

Despite the growing recognition of ncRNAs as potential therapeutic targets for PBC, several critical barriers impede their clinical translation. Firstly, the pleiotropic nature of ncRNAs complicates target specificity. For instance, miRNAs such as miR-506, which are upregulated in PBC and implicated in BEC dysfunction through different pathway modulation, often regulate multiple downstream effectors (58). This multi-target characteristic raises concerns about off-target effects and unpredictable systemic consequences when using RNA interference (RNAi) or antisense oligonucleotide (ASO) therapies. Secondly, effective delivery systems remain a bottleneck. Both nanoparticles and extracellular vesicles show therapeutic potential as ncRNA delivery vehicles, but critical challenges persist, including complexities in production standardization, biodistribution control, and biocompatibility (99). Safety concerns such as immunogenicity and off-target effects also require rigorous preclinical validation before clinical translation (100). Thirdly, interspecies heterogeneity in ncRNA networks limits preclinical predictability. Murine models of PBC fail to fully recapitulate human-specific lncRNA signatures associated with disease progression, such as the MALAT1/NF-kappaB regulatory axis identified in patient-derived organoids (101). Finally, regulatory challenges persist due to the lack of standardized biomarkers for monitoring ncRNA therapeutic efficacy. Current serum markers like alkaline phosphatase (ALP) correlate poorly with specific ncRNA activity, necessitating novel companion diagnostics.

### 4.3 Emerging opportunities

Next-generation technologies offer promising solutions. Single-cell RNA sequencing (scRNA-seq) can deconvolute ncRNA heterogeneity across immune subsets (102). Guo et al. (103) established the SCancerRNA database through scRNA-seq, creating a pivotal resource for detecting ncRNA biomarkers (lncRNAs, miRNAs, piRNAs, snoRNAs, circRNAs) at single-cell resolution, significantly advancing ncRNA-based diagnostic development, targeted therapy design, and computational prediction of diseases. In parallel, advancements in nascent RNA sequencing technologies (e.g., fastGRO) have further expanded the methodological repertoire for ncRNA discovery (104). The CRISPR/Cas system has emerged as a powerful genome-editing tool across various organisms, with early studies demonstrating its feasibility for knocking out non-coding genes in human cell lines (105). Recently, leveraging massively parallel CRISPR-Cas13 forward transcriptomic screens, researchers have overcome the limitations of DNA-based perturbation and identified a core set of essential lncRNAs (106). Artificial intelligence (AI) also holds potential: machine learning has been widely applied to predict ncRNA-protein interactions, and novel deep learning algorithms have been developed to infer miRNA-disease associations based on known lncRNA-miRNA interactions (107, 108).

While persistent knowledge gaps in ncRNA-PBC interplay intersect with emerging technological innovations, three synergistic priorities demand urgent attention. Foremost, establishing consensus protocols for ncRNA isolation and normalization in biofluids remains imperative for cross-study validation. Concurrently, organoid-based platforms can be developed for co-culturing patient-derived cholangiocytes with autologous immune cells to study ncRNA crosstalk. Translational efforts should prioritize ncRNA agents with dual biomarker-therapeutic roles, such as the HCV-validated miR-122 inhibitors Miravirsen (Santaris Pharma) and RG-101 (Regulus Therapeutics) – both demonstrating clinical-phase efficacy (Phase II/Ib) – might be rigorously repurposed for PBC after target validation (109). Addressing these priorities through multidisciplinary collaboration will accelerate the translation of ncRNA discoveries into clinical practice.

## 5 Conclusion

Primary biliary cholangitis represents a paradigm of autoimmune liver disease where ncRNAs serve as critical regulators interfacing female-skewed immunopathology, inflammatory cascades, BA metabolism, and gut-liver axis interactions. Emerging evidence highlights the pleiotropic involvement of miRNAs, lncRNAs, and circRNAs across the PBC continuum – from early bile duct injury to end-stage cirrhosis. Beyond mechanistic insights, these molecules possess transformative clinical potential: serum miR-122 and exosomal lncRNA H19 may soon complement AMAs as diagnostic biomarkers, while CRISPR-engineered circRNA sponges or nanoparticle-encapsulated antagomirs could pioneer RNA-based therapies for UDCA-non-responsive patients (81).

The ongoing revolution in ncRNA research is transforming the landscape of autoimmune disease investigation, refreshing our understanding of PBC pathogenesis and progression while paving the way for innovative therapeutic strategies. Nevertheless, realizing this potential demands a concerted effort to bridge existing gaps. Future studies must prioritize “human-centric” approaches by leveraging organoids, multi-omics cohorts, and AI-powered network pharmacology, with the goal of decoding the spatiotemporal dynamics of ncRNAs in PBC.
